# Polyclinic Notes

**Published:** 1909-10-16

**Authors:** 


### POLYCLINIC NOTES.

The opening lecture of the new session was delivered on Monday, October 4, by Dr. J. H. Nicoll, of Glasgow, on "The Treatment of Senile Enlargement of the Prostate." The College opened on September 13, and already a number of new subscribers have been enrolled, while the attendances at the Clinics and Practical Classes have been most encouraging. Any medical practitioner paying one guinea at the present date and entering his name as a subscriber will have the right to all the privileges of the Polyclinic until the end of the year 1910. A new course of Practical Classes will commence on Monday, November 1, and it is anticipated that the list of these classes will be considerably increased. The Council has made special arrangements to secure practical personal tuition in the use of such instruments as the ophthalmoscope, laryngoscope, otoscope, bronchoscope, cystoscope, sigmoidoscope, sphygmograph, sphygmomanometer, &c. Thus the wants of practitioners who have but a brief time in town, or who desire more individual opportunities than are possible in a class, can be satisfied. All communications should be addressed to Major Vint, M.B., 22 Chenies Street, Gower Street, W.C.

